# An Epidemiological and Economic Simulation Model to Evaluate Strategies for the Control of Bovine Virus Diarrhea in Germany

**DOI:** 10.3389/fvets.2019.00406

**Published:** 2019-11-19

**Authors:** Jörn Gethmann, Carolina Probst, Jason Bassett, Pascal Blunk, Philipp Hövel, Franz J. Conraths

**Affiliations:** ^1^Friedrich-Loeffler-Institut, Federal Research Institute for Animal Health, Institute of Epidemiology, Greifswald, Germany; ^2^Institut für Theoretische Physik, Technische Universität Berlin, Berlin, Germany; ^3^School of Mathematical Sciences, University College Cork, Cork, Ireland

**Keywords:** bovine viral diarrhea, disease control, economic analysis, cost-benefit analysis, agent-based model, dairy cattle

## Abstract

Models can be used to plan, evaluate, and improve programs for animal disease control. In Germany, a nationwide compulsory program to eradicate Bovine viral diarrhea (BVD) is in force since January 2011. As it is associated with substantial expenditures, the program is currently under revision. To provide the basis for a science-based decision on the future course of BVD control in Germany, we evaluated 13 scenarios (sc1-13) with respect to the chance of reaching freedom from disease and their economic implications for a period of 20 years (2011–2030). To simulate the impact of different control strategies on disease dynamics, a disease spread model was developed. To estimate the effects of a transient infection (TI) on animal level, a gross margin analysis was performed. To assess the value of cattle that died prematurely, a valuation model was used. Finally, an economic model was developed to perform a cost-benefit analysis and to compare each control scenario with a baseline setting with no BVD control. Costs comprised the expenditures for diagnostics, vaccination, preventive culling, and trade restrictions. Benefits were animal and production losses avoided by having control measures in place. The results show that reducing the PI prevalence on animal level to 0% is only feasible in scenarios that combine antigen or antibody testing with compulsory vaccination. All other scenarios, i.e., those based exclusively on a “test and cull” approach, including the current control program, will, according to the model, not achieve freedom of BVD by 2030. On the other hand, none of the scenarios that may lead to complete BVD eradication is economically attractive [benefit-cost ratio (BCR) between 0.64 and 0.94]. The average direct costs of BVD in Germany are estimated at 113 million Euros per year (34–402 million Euros), corresponding to 28.3 million Euros per million animals. Only the concepts of the former and the current national BVD control program (“ear tag testing and culling”) may reduce the BVD prevalence to 0.01% with an acceptable BCR (net present value of 222 and 238 million Euros, respectively, with a BCR of 1.22 and 1.24).

## Introduction

Bovine viral diarrhea (BVD) is an important infectious disease in cattle with a major economic impact that varies within and between countries (1, 2). Most of the economic damage is caused by a lower reproductive performance in dairy cattle, including reduced conception rate, abortion and reduced milk yield. Depending on the stage of pregnancy at the time of infection, vertical BVD transmission may result in abortion/stillbirth, congenital defects, growth retardation or the birth of persistently infected (PI) calves, which are often small and unthrifty, have increased susceptibility to other diseases and may eventually die from mucosal disease (3). Horizontal transmission can occur by direct or indirect contact with virus-shedding animals. The causative agent, BVD virus (BVDV), belongs to the genus Pestivirus of the family *Flaviviridae* and is divided into two genotypes: Pestivirus A (previously BVDV-1) and Pestivirus B (previously BVDV-2) (4). Although the existence of Pestivirus B has been confirmed in Germany (5), the predominant genotype in the country is Pestivirus A.

A number of countries in the European Union, e.g., Spain, do not monitor BVD at the national level (6). However, most countries have implemented voluntary or mandatory BVD control programs, which can lead to a significant decline in the PI prevalence (7). The programs differ in the way PI animals are detected, and in allowing or excluding vaccination. Some successful programs combine the test and cull strategy with vaccination, e.g., in Belgium, Ireland and Scotland (8). Others were successful without using vaccination, e.g., those implemented in Scandinavian countries, Austria and Switzerland (9–14). The economic impact of BVD and different control strategies has recently been reviewed in a number of publications (14–16).

According to the German statistical office (Destatis) and the Federal Office for Agriculture and Food, in 2017 there were 12.37 million heads of cattle in the country, including 4.2 million dairy cows, distributed in 143 thousand cattle farms. In Germany, the first voluntary BVD control program was developed in the late 1980s by the federal state of Lower Saxony (17). Central elements were the identification and elimination of PI animals and the systematic vaccination of all female offspring (18). Later, other federal states launched their own BVD control programs, on either a voluntary (e.g., Bavaria, North Rhine Westphalia, Lower Saxony) or a compulsory basis (e.g., Saxony-Anhalt) (19). However, the programs differed between the federal states and participation was at least in the beginning voluntary, with the consequence that little progress was achieved (20). There were also drawbacks in PI-free herds that had become seronegative and thus fully susceptible in an environment where infectious pressure of BVDV was high (21).

On 3 November 2004, BVD became a notifiable animal disease in Germany and on January 1, 2011, a nationwide compulsory BVD control program was started (22). At that time, the PI prevalence in Germany was at about 0.5% on animal level (23) and a limited proportion of the population was vaccinated against BVD. Consequently, all animals had to be tested for BVDV or its genome. The new regulations of 2011 introduced the testing of all newborn calves combining the cattle ear tag application with the sampling of a small ear tissue plug, which was subjected to BVDV testing (24). PI animals have to be eliminated (either immediately culled or slaughtered within seven days). It is also mandatory to test animals prior to movement if they have not been assigned a BVD status. Only cattle that have tested negative for BVDV (“unsuspicious animals”) may be traded. If an animal tests positive for BVDV, it is either removed (usually slaughtered) or retested after 6 weeks to rule out a transient infection. The German BVD control program has always allowed the use of vaccines, since they are considered a useful addition for preventing the formation of PI calves. In Germany, one modified live and several inactivated BVD vaccines are currently registered and can be used in different vaccination schemes. Since 2009, all BVD vaccinations and test results are recorded at the individual animal level in the national animal identification database (“*Herkunftssicherungs- und Informationssystem für Tiere,”* HIT), which is used for the registration of cattle holdings, all cattle individually, and for movements of cattle. Reliable BVD data are available since mid-2011. Based on the time point and the results of the BVD tests, an algorithm integrated into HIT calculates the individual BVD status: (i) without status; (ii) unsuspicious, i.e., antibody negative or mother of negative calf; (iii) first test positive; (iv) PI animal, i.e., two consecutive positive tests, or positive test without confirmation, or a calf of a PI mother.

Between the onset of mandatory testing in January 2011 and December 2016, more than 34 million animals were tested, registered in the HIT database and assigned a BVD status. The proportion of PI affected farms (animals) was reduced from 3.44% (0.48%) in 2011 to 0.16% (0.02%) in 2016. Although no recent studies are available, the seroprevalence in Germany is still assumed at 10-25%, depending on the region (25). The national BVD control strategy is currently under revision. The following alternatives to the current control policy are under discussion: (1) Stop BVD control and monitoring^1^ including a strict non-vaccination policy. (2) Continue controlling and monitoring BVD as laid down in the national regulation implemented from 2011 to 2016. (3) Proceed as in (2) with additional trade restrictions for BVD-affected cattle farms. (4) Continue BVD control as laid down in the current BVD regulation (antigen-detection by ear tag). (5) Proceed as in (4) with additional antibody testing (AbT) in individual or pooled serum/plasma samples from young stock between 9 and 12 months (so called “*Jungtierfenster”*) (26, 27) and voluntary vaccination. (6) Proceed as in (5) with additional compulsory vaccination. In this study, we evaluated 13 scenarios including the current BVD control policy, different combinations of the above-mentioned alternatives, and a baseline scenario (no BVD control) with respect to the chance of disease eradication and the economic implications in a period of 20 years (2011–2030).

## Materials and Methods

### Scenarios

Starting with the baseline scenario without any BVD control (sc1), we developed 12 alternative scenarios and simulated the course of BVD from 2011 onwards. In sc2, we simulated the former BVD control program that was in place in Germany 2011–2016. In sc3, we simulated the control program that is currently in place. In sc4, we simulated the immediate cessation of BVD control on July 1, 2017. The other nine scenarios were deduced from the current epidemiological situation and represent potential options for future BVD control based on various combinations of measures.

Each scenario is a chronological order of strategies; each strategy consists of a combination of measures (e.g., ear tag and vaccination; Table 1). All 13 scenarios start with the strategy “no BVD control” (sc1). By contrast, scenarios sc2-13 include the former BVD regulation as implemented on January 1, 2011. While sc2 represents only the continuation of the former BVD regulation (in place from 2011 to 2016), scenarios sc3-13 go beyond sc2 by including also the measures foreseen in the current BVD regulation as implemented on June 27, 2016. While sc3 represents the continuation of the current BVD regulation, sc4-13 implement different further strategies in the model from July 1, 2017 onwards.

**Table 1:** Overview of the 13 modeled BVD control scenarios.

**Scenario**	**Strategies (start)**				**Measures**	
	**24/11/1983**	**01/01/2011**	**29/06/2016**	**01/07/2017**		
1	1				No control	
2	1	2			Former regulation (ear tag)	
3	1	2	3		New regulation (ear tag, trade restrictions)	
4	1	2	3	1	No control	
5	1	2	3	4	Ear tag, compulsory vaccination	
6	1	2	3	5	Ear tag	AbT 2×/year
7	1	2	3			AbT 1×/year
8	1	2	3	6	Ear tag, compulsory vaccination	AbT 2×/year
9	1	2	3			AbT 1×/year
10	1	2	3	7		AbT 2×/year
11	1	2	3	8	Compulsory vaccination, stop testing	Inmediate stop
12	1	2	3			Slow stop
13	1	2	3	9	Compulsory vaccination	AbT 2×/year

*The different colors represent different strategies. Each scenario is a chronological order of strategies; each strategy consists of a combination of measures*.

**Scenario 1** (sc1) reflects the basic situation, in which no control program is in place, i.e., no efforts are made to detect or remove PI animals. Thus, no vaccination expenses or other preventive expenditures are included in the economic model. This scenario is hypothetical, since Germany has implemented BVD control measures for a long time. Scenario sc1 was included to compare different intervention scenarios with a “baseline” scenario that does not include any intervention.

**Scenario 2** (sc2) was designed according to the control program that was in place in Germany between 2011 and 2016 (former BVD regulation). This program included obligatory antigen screening (ear tag) in newborn calves (<7 days) and the removal of PI animals within 60 days after confirmation of a BVD positive test result. The strategy also included voluntary vaccination and individual antigen testing in adult animals before trading them.

**Scenario 3** (sc3) combines the strategy of the former BVD regulation with the control program, which is in place in Germany since June 27, 2016 (new BVD regulation). Similar to sc2, it includes antigen screening (ear tag) in newborn calves (<7 days) as well as individual antigen testing in adult animals before trading and voluntary vaccination. In contrast to sc2, sc3 includes the removal of PI animals within 40 days (instead of 60 days in sc2) and a trade restriction of 40 days for farms after confirmation of a BVD infection in the herd.

**Scenario 4** (sc4) is similar to sc3 but assumes that BVD control has stopped on July 1, 2017.

**Scenario 5** (sc5) is similar to sc3 with the only difference that it includes compulsory vaccination starting on July 1, 2017.

**Scenarios 6 and 7** (sc6, sc7) include antigen detection after birth or before trade (similar to sc2, sc3, and sc5). In addition, they also include AbT, either twice a year (sc6) or once a year (sc7) and PI removal within 40 days as well as trade restrictions of 40 days, but no vaccination.

**Scenarios 8 and 9** (sc8, sc9) are similar to sc6 and sc7 and include AbT, either twice a year (sc8) or once a year (sc9), but in contrast to sc6 and sc7, they include compulsory vaccination.

**Scenario 10** (sc10) only includes AbT twice a year.

**Scenarios 11 and 12** (sc11, sc12) represent the decision to stop testing on July 1, 2017 and to switch to vaccination only, either immediately (sc11) or after a transitional period (sc12).

**Scenario 13** (sc13) combines the measures of AbT twice a year and compulsory vaccination. Testing of ear tags plugs is stopped after a transition period of 1 year (06/2018) to allow the whole cattle population to become protected by vaccination against BVD.

### Models

The evaluation of the eradication success and profitability of different BVD control scenarios was done in four steps. (1) An agent-based disease spread model (DSM) was developed to simulate the dynamics of BVD spread and immunity within the population. (2) A gross margin analysis (GMA) was performed to estimate the economic impact of a transient BVD infection. (3) To estimate the value of PI animals that prematurely die of BVD, we developed a stochastic animal valuation model (AVM). (4) We developed an economic model and performed a cost-benefit analysis (CBA) using the results of the DSM, GMA and AVM.

#### Disease Spread Model (DSM)

To simulate the dynamics of BVD in different scenarios, a stochastic, event-driven, hierarchical agent-based disease spread model (DSM) was developed. Within the DSM, trade was realized using a farm manager and a market. The farm manager keeps the size of the farms constant. When a farm has too many animals, it will sell animals to a market, if it needs animals it will buy animals from the market. These movements are based on trade criteria. If there is no demand on animals with a certain criteria, they will be slaughtered. Whenever there are not enough animals in the market, new animals are created (simulation of imports from EU member states). The DSM takes into account (i) five individual disease status, namely susceptible, transiently infected (TI), persistently infected (PI), recovered from transient infection, and vaccinated; (ii) disease transmission between animals, (iii) disease transmission (trade) between farms, and (iv) the introduction of new animals into the population. We assume that (1) recovery from natural BVD infection leads to lifelong immunity, that (2) calves with maternal antibodies are protected for about 6–9 months after birth and that (3) vaccination requires yearly boosting. To take the constant risk of disease introduction through imports into account, the model includes a trade manager and the PI prevalence of imported animals was set at 2%. Each individual animal is simulated from birth to death.

The following input parameters were retrieved from the DSM:

- Number of farms with no active infection (i.e., all animals susceptible), with protected (recovered or vaccinated) animals, and with PI animals per scenario and year;
- Number of PIs and TIs and animals that died from mucosal disease per scenario and year;
- Number of farms and animals subject to control measures, i.e., number of diagnostic tests (ear tag, blood samples for PCR and AbT), scenario and year; number of vaccinated animals and number of vaccinations by scenario and year;

The following values from the German cattle trade database (HIT) were used as input parameters: Number of farms and farm size distribution, age distribution for males and females, cause of death, age at first calving, calving interval, BVD test results, and number of PIs and TIs between 2010 and 2017.

The simulation was run in C++ for the years 1983–2030 (total of 20,000 days). Thereof, the first 10,000 days were used to reach a stable state in disease dynamics. After 5,000 days an equilibrium between susceptible, recovered, PI and TI animals was reached. The source code of the model can be accessed in a repository (https://github.com/Yperidis/bvd_agent_based_model). The disease spread model is described according to Grimm et al. (28) and can be found at arXiv (29). Further details on the model and its validation can be found at Bassett (30). Since our statistical software is not equipped to handle extremely large datasets (12.4 million head of cattle, 13 different scenarios), we first run the model on a subset of 360,000 animals. We then scaled the results up to the whole cattle population in Germany using a factor of 39.2.

#### Gross Margin Analysis (GMA)

To estimate the effects of a transient BVD infection in terms of production losses, a gross margin analysis (GMA) on animal level was performed for dairy cows and heifers. To consider the heterogeneity of cattle farms in Germany in terms of herd size and management, the GMA was performed as a stochastic model. All estimations are based on data of the German Association for Technology and Structures in Agriculture (“*Kuratorium für Technik und Bauwesen in der Landwirtschaft e.V., KTBL”*), the animal health services (“*Tiergesundheitsdienste”*), animal disease compensation funds of the federal states (“*Tierseuchenkassen*“), Destatis, and HIT database (Table S1). The GMA was carried out with R statistical software (31) and the packages *xlsx, ggplot2, sm, fitdistrplus, MASS, foreign, EnvStats, stats, graphics, utils*, and *base*. For each set of parameters, 10,000 iterations were conducted.

We calculated the gross margin (GM) for both, healthy animals (GM_h_), i.e., in absence of BVD, and for cattle with a transient BVD infection (GM_TI_) by adapting the respective variables for the calving interval, milk yield, and veterinary costs. Direct costs incurred by a TI animal (DC_TI_) were then calculated as the difference between both:

$$\begin{array}{l} DC_{TI}=GM_{h}-GM_{TI} \end{array}$$

To quantify the average impact of TI on reproduction, we used the calving interval (Ci). In case of BVD induced abortion/stillbirth, the Ci was increased by a certain number of days.

To quantify these, we first estimated the probability of the outcome (abortion/stillbirth) on five different time periods, including four pregnancy stages (days 1–70; 71–120; 121–180; 181–285) and the post-partum stage (days 286–385), based on Viet (32). We then calculated the overall probability for an animal being in a particular stage with a specific outcome (birth of a PI calf, abortion/stillbirth, congenital defect/growth retardation, birth of an immune calf, no influence; Table S5). Finally, we estimated the number of days the “healthy” Ci had been prolonged in each stage, as the average of the respective period (Table S6).

To quantify the impact of TI on the revenues in milk sale, we multiplied the difference between the milk yield of a “healthy” and that of an infected cow with the average milk price. All equations and input parameters are shown in Table S1.

#### Animal Valuation Model (AVM)

To estimate the market value (v_c_) of PI animals that prematurely die of BVD, we devised a stochastic animal valuation model (AVM) based on the appraisal guidelines of the animal health compensation fund of North-Rhine-Westphalia governing indemnity payments for livestock (“*Schätzrahmen*”). Two different equations were used (for dairy cows as well as calves and young stock, respectively). The equations combine the age of the animal (in months) with production, reproduction and animal health data (Table S2). The AVM was carried out using R statistical software (31) using the packages *xlsx, pander, ggplot2, sm, fitdistrplus, MASS, foreign, EnvStats, stats, graphics, utils*, and *base*.

#### Economic Model

To simulate the economic impact of BVD and the control measures on national level, we developed a stochastic economic model in @Risk for Microsoft Excel 2010 with 20,000 iterations, covering a period of 20 years (2011–2030). For parametrization, the results of the DSM, GMA, and AVM were used. For all other parameters, we used empirical distributions based on literature and expert opinion (Horst Schirrmeier, Kerstin Wernike, and Martin Beer from the FLI, Georg Wolf from the Ludwig-Maximilians-Universität München, and Karsten Donat from the animal disease compensation fund Thüringen). All parameters and equations are listed in Tables S3, S4. We differentiated between four different age groups: calves (1–6 months), young stock (7–18 months), heifers (females 19–28 months), and cows (females >28 months).

##### Total costs of BVD

The total costs of each scenario for the 20-year study period (2011–2030) include direct and indirect costs.

*Direct costs PIs*. If not discovered in time, PIs may display clinical symptoms thus requiring veterinary treatment, and they usually die within the first 2 years of life (33). Hence, the direct costs incurred by a PI animal include veterinary costs and costs incurred through premature death. The latter include the disposal costs for a calf, young stock or cow, and the lost market value of the animal (calculated in the animal valuation model, see chapter 3.2.3). The costs of culling or preventive slaughter were included in the indirect costs (as they represent a BVD control measure).

*Direct costs Tis*. Transient infection with BVD may cause production losses in all age groups. In calves and young stock, losses include poor growth and weight loss, and were estimated using a risk uniform distribution (Table S1). For heifers, losses include increased calving interval and were estimated in the GMA for heifers (Table S1). For cows, losses include increased calving interval (Ci) and the reduced milk yield. They were estimated in the GMA for cows (Table S1). For heifers and cows, we took a sample from the GMA results using the RiskResample function in @Risk.

*Indirect costs of BVD*. Depending on the scenario, the indirect costs of BVD include the costs to prevent infection, i.e., diagnostic measures and vaccination, as well as costs to control the disease, including culling or preventive slaughter of PIs and trade restrictions.

Diagnostic measures: Individual BVD diagnosis can either be done through antigen or BVDV genome detection (tissue sampling by ear tag in calves or blood sampling in adults) or AbT (blood sampling in young stock). Ear tags are applied by the farmer. Hence, the costs for antigen detection in ear tags include material (ear tags and a certain percentage of ear tag pliers) and labor (shipping, testing and communicating test results). In contrast to tissue samples, blood samples need to be taken by a veterinarian. So at individual animal level, the costs for antigen detection in blood include the costs for blood sampling and testing (PCR). On the farm level, the costs include the herd fee, handling, shipping, and communicating test results. Similar to blood sampling for antigen detection, the costs for blood sampling for AbT include on the farm level the herd fee, handling, shipping, and communicating results. At the individual animal level, they include costs for sampling and testing (ELISA).

Vaccination: In all six scenarios that include vaccination (sc5, 8, 9, 11, 12, 13), vaccination was planned to be compulsory for all female animals before getting pregnant. In the remaining seven scenarios, vaccination was not included. Depending on the vaccination scheme, several immunizations are required. As vaccines must only be administered by veterinarians in Germany, vaccination costs include the herd fee charged by the veterinarian. At the individual animal level, we accounted for the number of immunizations per animal and the costs for the vaccine and vaccination.

Preventive slaughter of PIs: Usually, PIs are culled or preventively slaughtered as soon as they are discovered. The lost revenues and costs were calculated by multiplying the number of preventively slaughtered PI calves, young stock and cows by their relative market value, which was assumed to be lower than the value of an average slaughter animal.

Trade restrictions: If a BVD infected animal is detected, cattle must not be moved from the affected premise for 40 days according to national legislation. Non-pregnant cattle can only leave the farm for slaughter or if the animal has been subjected to a second test 40 days after the initial analysis at the latest. Pregnant cattle may be moved if the animal has been subjected to a serological test after the 150th day of gestation with a negative result. We assumed that each affected farm would move three pregnant and three non-pregnant dams within 40 days of quarantine (34). This implies the following costs for these three pregnant and three non-pregnant animals: travel 10 €, taking blood samples 10 €, handling and shipping samples 9 €, laboratory analysis 30 € (3 × 10 €). Hence, the movement ban would result in 118 € (2 × 59 €) additional veterinary costs per affected premise.

##### Cost-benefit analysis

A cost-benefit analysis (CBA) was conducted to evaluate the profitability of each scenario compared with sc1 (no BVD control) throughout the study period (2011–2030). The CBA was performed as described by Rushton et al. (35). It was conducted as a stochastic simulation with the add-on @Risk 5.7 (Palisade Corporation, Ithaca, NY, USA), performed with 10,000 iterations. The built-in @Risk sensitivity analysis tool was used to evaluate which input parameters had the strongest effect on the results. The benefit-cost ratio of each scenario (BCR_s_) was calculated by dividing the present value of benefits (PVB_s_) by the present value of indirect costs (PVIC_s_).

$$\begin{array}{l} BCR_{s}=\frac{PVB_{s}}{PVIC_{s}} \end{array}$$

PVB and PVIC were calculated as follows (where r is the interest rate):

$$\begin{array}{l} PVB_{s}=\overset{2030}{\underset{y=2011}{\sum }}\frac{B_{y,s}}{{\left(1+r\right)}^{\left(y-2011\right)}}\\ PVIC_{s}=\overset{2030}{\underset{y=2011}{\sum }}\frac{IC_{y,s}}{{\left(1+r\right)}^{\left(y-2011\right)}} \end{array}$$

The annual benefit (B_y, s_) of each scenario was calculated as the difference in disease (i.e., direct) costs between sc1 (DC_y, s1_) and the respective alternative scenario x (DC_y, s_).

$$\begin{array}{l} B_{y,s}=DC_{y,s1}-DC_{y,s} \end{array}$$

All monetary values were expressed in Euros. Break-even points (the point at which total cost and total revenue are equal) were obtained from the annual results for each scenario. The analytical objective of this part of the study was to estimate the net present value (NPV) of different scenarios over the study period of 20 years. To enable a comparison between past, current and future values, all monetary flows for the benefits and costs were discounted at a rate of 3%. For reasons of comparability, foreign currencies referred to in the international literature were converted into Euros using the average currency exchange rate valid in the year of publication of the respective study.

## Results

### Disease Spread Model

**Figure 1:**
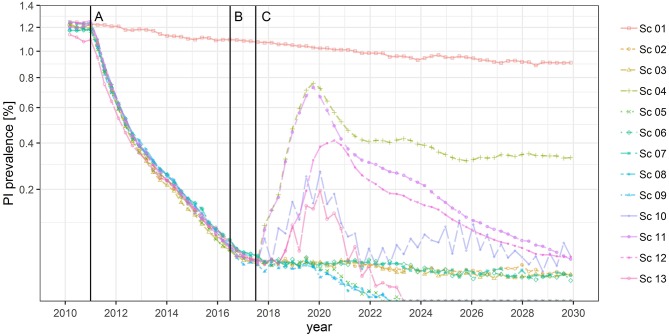
Predicted PI prevalence between 2010 and 2030 for each BVD scenario. **(A)** Start of the former BVD regulation (sc2). **(B)** Start of new BVD regulation (sc3). **(C)** Start of alternative scenarios (sc4–sc13).

The results of the DSM have been described elsewhere (30). According to the DSM, only scenarios that combine antigen or antibody testing with compulsory vaccination (sc5, sc8, sc9, sc13) are likely to reduce the PI prevalence on animal level to 0%, i.e., may lead to the eradication of BVD (Figure 1). In sc5, the PI prevalence will reach 0% in the third quarter of 2022, in sc8 and sc9 in the fourth quarter of 2022, and in sc13 in the second quarter of 2023. In scenarios that include compulsory vaccination, the prevalence of protected animals (recovered or vaccinated) will be above 75% by 2030. Scenarios that include antigen testing (sc2, sc3, sc6, sc7) may reduce the PI prevalence to a value 0.01%. All other scenarios, including the control program currently in place, are unlikely to lead to BVD eradication. In scenarios sc4, sc10, sc11, and sc12, the PI prevalence is predicted to stay in the range of 0.01–0.05% by 2030. In scenario sc1, the PI prevalence will decrease gradually from 1.2 to 0.9% and the seroprevalence (recovered animals) will go down from 64 to 47%, while the proportion of susceptible animals will increase from 35 to 52% and the TI prevalence is predicted to stay nearly constant at a level of 0.25–0.33%.

As soon as testing stops in scenario sc4, the PI prevalence will rapidly rise up to 0.75% in 2019 and is predicted to decrease then again, but a proportion of 0.33% PI animals will still remain by 2030 (Figure 1, Table 2). Although scenarios sc11 and sc12 include vaccination, they do not combine it with testing, and will not lead to BVD eradication according to the model predictions. In all other scenarios, the prevalence of protected animals will be 3–7% by 2030.

**Table 2:** Prevalence of PI and BVD antibody-positive animals (recovered or vaccinated).

**Scenarios**	**Prevalence in 2030 (%)**	
	**PIs**	**Antibody positive animals**
Scenario 01	0.894	46.91
Scenario 02	0.010	4.36
Scenario 03	0.009	3.17
Scenario 04	0.317	18.93
Scenario 05*	0.000	75.49
Scenario 06	0.011	3.49
Scenario 07	0.010	3.82
Scenario 08*	0.000	75.59
Scenario 09*	0.000	75.61
Scenario 10	0.053	6.53
Scenario 11*	0.026	75.95
Scenario 12*	0.026	75.98
Scenario 13*	0.000	75.62

Scenarios that include vaccination are marked with an asterisk (*).

### Gross Margin Analysis

**Figure 2:**
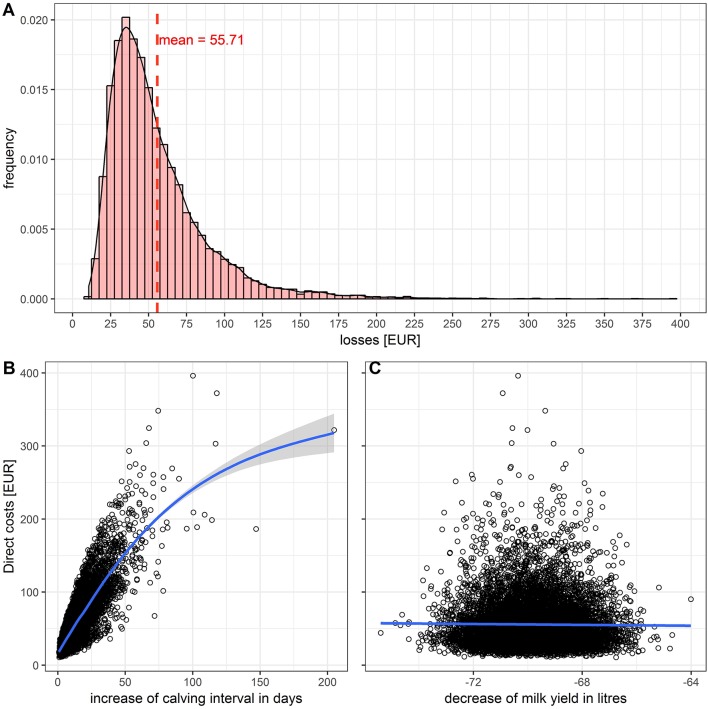
Impact of a transient BVD infection in a dairy cow: **(A)** Histogram of the total losses. **(B)** Influence of the increased calving interval. **(C)** Influence of the decreased milk yield on the losses.

In cows, the direct costs of a transient infection were estimated at 55.71 Euros per animal on average (Figure 2A). An increased Ci of 0–60 days increased the direct costs by 0–180 Euros (Figure 2B). A reduced milk yield of 64–76 L reduced the GM in average by 50 Euros (Figure 2C). In heifers, the average costs were estimated at 7.80 Euros, and for calves and young cattle between 0 and 10 Euros (mean 5 Euros).

### Animal Valuation Model

The results of the AVM are shown in Table 3. The mean value of a cow was estimated at 1,451 Euros (Table 3).

**Table 3:** Estimated market value of cattle of different age classes.

	**Calves (0–6 months)**		**Young stock (7–24 months)**		**Heifers**						**Cows**
**Age in months**	**1**	**···**	**12**	**···**	**24**	**25**	**26**	**27**	**28**	**29**	
Mean	304.4	···	902.3	···	1,616.8	1,673.4	1,729.9	1,855.1	1,913.9	1,972.6	1,451.4
Min	165.1	···	489.3	···	876.7	907.4	938.0	1,005.9	1,037.8	1,069.6	376.9
1st Qu	276.6	···	819.9	···	1,469.1	1,520.5	1,571.9	1,685.7	1,739.0	1,792.4	1,260.8
Median	316.2	···	937.4	···	1,679.7	1,738.4	1,797.1	1,927.2	1,988.2	2,049.2	1,493.8
3rd Qu	339.8	···	1,007.4	···	1,805.0	1,868.1	1,931.2	2,071.0	2,136.6	2,202.1	1,682.6
Max	358.6	···	1,062.9	···	1,904.5	1,971.1	2,037.7	2,185.2	2,254.4	2,323.5	1,936.3

### Economic Model

The results of the economic model are listed in Table S7 (direct, indirect, and total costs per year). Direct costs are estimated at 113 (34–402) million Euros per year. They are predicted to stay nearly constant over time, since the PI prevalence decreases very slowly. Table 4 details the minimum, maximum, and mean total costs. The total costs arising from BVD in sc1 (no BVD control) were estimated at 2.258 billion Euros. The least expensive scenarios among those that continue with BVD control are sc12 and sc11 (stop testing and switch to vaccination, either gradually or immediately), followed by sc3 (ear tag and quarantine), and sc2 (ear tag). Scenarios sc11 and sc12 generate total costs of 1.81 and 1.77 billion Euros, respectively. Similar to scenario sc4, the majority of costs are predicted to arise in the initial years, while from 2023 onwards both scenarios, sc11 and sc12, will become cheaper until they are expected to level off at about 65 and 66 million Euros per year. Sc2 and sc3 are predicted to cause total costs of 1.93 and 1.91 billion Euros, respectively. The costs of both scenarios will level off from 2017 onwards and lead to nearly constant sums of about 84 and 82 million Euros per year.

**Table 4:** Total costs (direct and indirect costs) per scenario (million Euros) with minimum and maximum values.

**Scenario**	**Mean**	**Minimum**	**Maximum**
sc1	2,258	1,821	3,497
sc2	1,933	1,614	2,540
sc3	1,909	1,602	2,469
sc4	1,413	1,162	2,195
sc5	2,525	2,167	3,139
sc6	3,655	2,966	4,520
sc7	2,511	2,122	3,214
sc8	7,229	5,567	9,128
sc9	2,703	2,308	3,287
sc10	2,680	2,094	3,488
sc11	1,812	1,503	2,402
sc12	1,769	1,430	2,534
sc13	2,747	2,206	3,498

**Figure 3:**
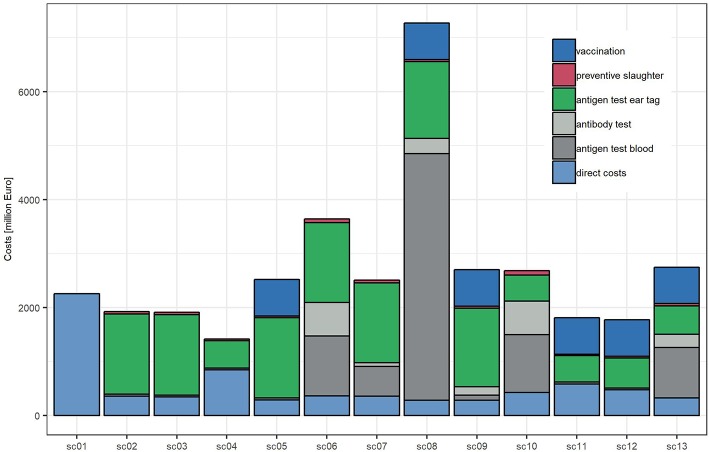
Direct and indirect costs incurred 2011–2030 in the 13 simulated scenarios.

Seven scenarios are predicted to be more expensive than sc1. The five most expensive scenarios all include AbT. With total costs of 7.23 billion Euros, the most expensive scenario will be sc8 (ear tag, AbT twice a year and vaccination). From 2017 onwards, sc8 is predicted to generate increasing costs until 2024, and from 2024 onwards, they will level off at 538 million Euros per year. As the PI prevalence is predicted to decrease to almost 0%, all costs are allocated to control measures, in particular antigen testing in blood (72%), antigen testing in ear tags (13%), vaccination (11%), and antibody testing (4%) (Figure 3).

The second most expensive scenario is sc6 (ear tag and AbT twice a year), which is predicted to generate total costs of 3.65 billion Euros, of which 90% (3.30 billion) are allocated to disease control. The costs of sc6, sc10, and sc7 peak in 2018 and then almost constantly decrease until 2030. Scenario sc13 (AbT twice a year, vaccination) is the third most expensive scenario and is expected to generate 2.75 billion Euros costs (thereof 88% for disease control), followed by sc9 (ear tag, AbT once a year, vaccination, 2.7 billion Euros), sc10 (AbT twice a year, 2.68 billion Euros), sc5 (ear tag, vaccination, 2.53 billion Euros), and sc7 (ear tag, AbT one a year, 2.51 billion Euros).

Toward the end of the study period, the yearly costs of all scenarios are predicted to level off at almost constant sums: The cheapest scenario will again be scenario sc4 (36% of sc1), followed by sc12, sc11, sc3, and sc2 (66–80% of sc1). Scenarios sc7 and sc10 will be slightly more expensive (103–104% of sc1), followed by sc5, sc9, sc13, and sc6 (138–171% of sc1), and sc8 is expected to stay the most expensive scenario (535% of sc1).

The highest disease impact is predicted to occur in sc1 and sc4, i.e., in scenarios with no control or stopping control measures altogether (2.26 billion Euros, 100% of the total costs of sc1, and 845 million Euros, 60% of the total costs of sc4). Among the scenarios with control, the highest disease impact is predicted to occur in sc11 and sc12 (584 and 473 million Euros, 32 and 27% of the total costs, respectively), followed by sc10 (427 million Euros, 16%) and sc2, sc6, sc7, sc3, and sc13 (360–324 million Euros, 10–19%). The lowest disease impact is expected to occur in sc5, sc8, and sc9 (280–285 million Euros, 4–11% of the total costs). All estimates are reported as medians.

Sensitivity analysis showed that the costs for AbT had the highest impact on the indirect costs. In scenarios without vaccination (sc6, sc7, sc10), the number of AbT will stay constant (~2.8 million tests per year for sc6 and sc10 and 1.4 million tests per year for sc7). On the other hand, the number of positive AbT is predicted to decrease to about 8% in scenarios sc6 and sc7, and to 12% in scenario sc10. In scenarios with vaccination (sc8, sc9, and sc13), the number of AbT will first decrease and stay constant from 2023 onwards (~475.000–946.000 tests per year). In scenarios with vaccination (sc8, sc9, sc13), the number of BVD antibody-positive animals will remain in the range of 132,000–265,000 from 2022 onwards.

Compared to sc1, the break-even points of all scenarios were estimated to be reached in 2017. However, only sc2, sc3, sc4, sc11, and sc12 are predicted to stay beneficial, while the cumulative costs of all other scenarios will rise in the following years and will finally be higher than the benefit. Scenarios sc6, sc7, sc8, and sc10 are unlikely to result in profit from 2018 onwards, sc9 from 2021, sc13 from 2022, and sc5 from 2023 onwards.

Table 5 lists the undiscounted and discounted benefit (B), indirect costs (IC), benefit-cost ratio (BCR), net value (NV), and net present value (NPV) per scenario.

**Table 5:** (A) Undiscounted and (B) discounted benefit (B/PVB), indirect cost (IC/PVIC), benefit-cost ratio (BCR), and net value (NV/NPV) of the 13 simulated scenarios (in million Euros).

**Scenario**	**(A) Undiscounted**				**(B) Discounted**			
	**B**	**IC**	**BCR**	**NV**	**PVB**	**PVIC**	**BCR**	**NPV**
sc1								
sc2	1.897	1.573	1,22	324	1.437	1.216	1,18	222
sc3	1.915	1.566	1,24	349	1.449	1.211	1,2	238
sc4	1.413	569	1,1	844	1.097	524	2,09	572
sc5	1.972	2.240	0,92	−268	1.488	1.658	0,9	−170
sc6	1.898	3.295	0,69	−1.397	1.437	2.433	0,59	−996
sc7	1.901	2.154	0,92	−253	1.439	1.633	0,88	−193
sc8	1.976	6.947	0,52	−4.971	1.491	4.837	0,31	−3.346
sc9	1.978	2.423	0,87	−445	1.492	1.785	0,84	−293
sc10	1.831	2.253	0,93	−423	1.388	1.710	0,81	−322
sc11	1.674	1.229	1,95	445	1.266	959	1,32	307
sc12	1.785	1.296	1,51	489	1.351	1.014	1,33	337
sc13	1.934	2.424	0,94	−490	1.458	1.772	0,82	−314

*The discounting rate is 3%*.

The discounted BCR was estimated to range between 0.31 (sc8) and 2.08 (sc4). It was >1 for scenarios sc2, sc3, sc4, sc11, and sc12. This means in case of scenario sc4, that 2.08 Euros are saved for each invested Euro, whereas only 0.31 Euros per invested Euro are saved in scenario sc8.

## Discussion

We developed two models (DSM and an economic model) to plan, evaluate, and improve BVD control programs. The models were applied to the situation in Germany, a country that is currently in the process of optimizing its BVD control strategy. Both models may also be applied in other countries. They can help to design or improve other disease control programs and to avoid problems that may be expected in their course.

### Economic Model

We estimated the direct costs of BVD to range from 34 to 402 million Euros per year in Germany, with a mean of 113 million Euros, corresponding to 28.3 million Euros per million animals. These results fall within the range of estimates that were previously obtained for other countries. Projected on costs per million animals, direct BVD costs were estimated at 18–21 million Euros for Switzerland (36), at 16.3 million Euros for Norway (37), at 10.3–28 million Euros for the Netherlands (15), and between 32 million (suckler cows) and 63 million Euros (dairy cows) for Ireland (38). High costs associated with BVD infection has led to increased disease in the cattle industry and to public eradication efforts. Although the German program has been regarded as successful in recent years (23), it is currently under revision as eradication has not yet been achieved.

All scenarios that include only “test and cull” strategies, i.e., also the current control program, are unlikely to have eradicated BVD in Germany by 2030. Only scenarios that combine either antigen or antibody testing with compulsory vaccination (sc5, 8, 9, 13) are likely to reduce the PI prevalence to 0% according to the model predictions. However, from an economic perspective, these scenarios are not beneficial.

Scenarios that combine antibody and ear tag testing (sc6, 7, 8, 9) may result in a significantly faster decrease of PI prevalence, but none of them is economically attractive due to the large numbers of tests required for surveillance. Scenario sc10 (AbT only) leads to an increase in PI prevalence and causes higher costs than sc1. In this case, risk-based surveillance may reduce the number of samples, while providing a high sensitivity at the same time (36). Risk-based categorization of farms could be performed by taking for example the number of animal movements and the disease status of the origin of purchased animals into account.

Of all simulated scenarios, only four were found to be economically attractive, namely scenarios sc2, sc3, sc11, and sc12, with an NPV of 222, 238, 307, or 337 million Euros, respectively. Although scenario sc4 has an NPV of 572 million Euros, it must be assumed that the disease costs will rapidly increase again if all control measures are abandoned. With scenarios sc11 and 12 (vaccination only), eradication does not appear to be feasible. If BVD control is stopped before the last PI is removed (see sc4), the PI prevalence is predicted to level off on the long term at about 0.33%. Previous studies do not advise premature discontinuation of control efforts, as a mainly seronegative cattle population is fully susceptible to BVD (39). Abandoning the long-standing goal of eradicating BVD may lead to necessity of imposing trade restrictions and, more importantly, to the loss of credibility of official disease eradication programs.

In summary, scenarios sc2 and sc3 were predicted to be successful in terms of both, disease eradication and benefit-cost ratio. However, a major current challenge for BVD eradication is the unrecognized import of inapparent or subclinical PI animals. A recent risk assessment showed that BVD is regularly introduced in the Netherlands through cattle importations and estimated that 334 cattle herds may become infected per year (40).

According to our results, the control program currently implemented in Germany is beneficial (BCR = 1.2). This is in line with calculations for Ireland (38) and Switzerland (36), which run similar control programs as Germany. Both groups calculated a higher BCR (10 and 1.9) than we did. In the Netherlands (15), where a control program based on bulk milk testing is in place, the BCR = 1.5 is slightly higher than the one we calculated for Germany. Other studies came to a negative BCR, e.g., for Styria, Austria, with a BCR of 0.83 (41). Comparing our results directly with those of others is difficult as the control programs are different, the assessment periods vary in time and duration, and the cattle structure (e.g., herd structure, trade patterns and animal density) may not be comparable. A recent review has only identified four countries (Norway, Ireland, France and Switzerland), where the implementation of BVD mitigation activities appeared economically justified after a specific period (16).

### Disease Spread Model

In scenario sc1, BVD reaches an endemic status with a PI prevalence of at least 0.9%. Over the study period, the PI prevalence was predicted to decrease slightly and no steady state was reached within the projected period, although the rate of reduction was very small. Most probably, this is due to fact that the disease transmission rates between animals and farms were estimated rather low, although they were based on values obtained from literature (32).

In scenario sc4, we observed a rapid increase of the PI prevalence up to 0.75% just after stopping BVD control, before it decreases and levels off at about 0.33%. The initial increase of the PI prevalence is due to the high number of naïve animals at the end of the control program. In the following years, the number of animals recovered from transient infection increases and the number of PI animals decreases until a steady state is reached. In contrast to sc1, the PI prevalence is lower, probably because in sc1 no steady state is reached.

**Figure 4:**
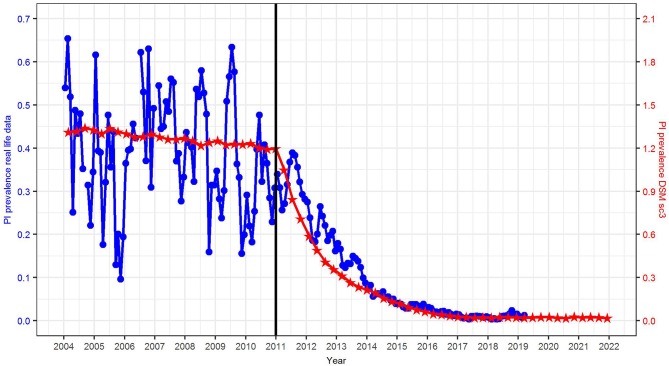
Comparison of the PI prevalence in reality (source: HIT, blue circles, left axis) and simulated in the DSM, scenario sc3 (red stars, right axis).

Compared to real life data, the initial PI prevalence in the DSM was three times higher (1.2 vs. 0.4%) (Figure 4). To explain this phenomenon, it has to be taken into account that (i) reliable data from the HIT database were not available until mid-2011 and (ii) in reality, several federal states had already implemented different types of voluntary control programs in 2011. Hence, the reported prevalence at the animal and farm level in previous years does not necessarily reflect a baseline scenario (without BVD control), but rather a heterogeneous situation. Compared with studies carried out prior the start of any control program, e.g., 2.1% in Lower Saxony, Germany (42) or 0.8–1.3% in Switzerland (9, 11, 43), the assumed PI prevalence of 1.2% in the DSM seems realistic.

Previous studies revealed BVD seroprevalences of 64–97% in Germany (44), 57.6% in Switzerland (45), and 33–54% in Belgium (46, 47). Compared with these studies, the antibody prevalence of 47% in the DSM seems to be realistic.

Compared with real data obtained from the HIT database, the reduction in PI prevalence seems to be predicted in a rather realistic fashion by scenario sc3, with the only difference that the PI prevalence was reduced to 0.01% in the first quarter of 2017 in reality, while this prevalence level will not be reached until 2024 according to the model prediction (Figure 4). This may be due to the fact that (i) the initial PI prevalence in scenario sc3 is three times higher than in reality, and (ii) we did not simulate further effects in the DSM, e.g., additional measures carried out by the farmers.

Scenarios that include only ear tag testing (sc2, sc3), only AbT (sc10) or a combination of both (sc6, sc7) will not lead to BVD eradication. Regarding scenarios sc2 and sc3, the reasons are imperfect tests, the continuous importation of PI animals, delayed removal of PI animals, and failure to take transient infections into account (23).

In scenario sc10, the number of PI animals increased after switching from ear tag to antibody testing only. This confirms experiences made in Switzerland in 2012/2013 (https://www.infosm.blv.admin.ch/public/). Hence, in terms of PI prevalence, this option is worse compared to ear tag testing (sc2, 3) Only scenarios that combine vaccination with either ear tag (sc5) or AbT (sc13) or both (sc8, sc9) are expected to lead to eradication according the modeling results. All scenarios that include AbT are predicted to lead to an unexpectedly high number of blood antigen tests. This is due to the fact, that, according to German BVD regulation, all cattle in a farm are subjected to a virus isolation test to detect viremic animals if an animal is confirmed antibody positive. Furthermore, maternal BVD antibody titers are still high enough to be detectable in calves until the age of 9 months. This can explain why so many animals (tested at the age of 7–18 months) were antibody positive in the DSM and why strategies that include AbT are so effective in reducing the PI prevalence. However, in reality, the number of antigen tests may be substantially lower, probably because animals subjected to AbT are older than 7 months. Moreover, it seems unlikely that all animals of a farm will be tested if a single BVD-positive result is obtained in this herd.

Other studies on the spread of BVD and the effect of different control strategies were published for Scotland (48), Ireland (49), and Italy (50). Nevertheless, the authors used other approaches to model disease spread or tested different control strategies. Tinsley et al. (48) compared only trade restrictions, using a network model. Thulke et al. (49) tested the change from the current control system (ear tag testing), which is similar to the German strategy, to serological testing. They also included additional factors, e.g., difficulties in changing the strategy. Iotti et al. (50) used a more general approach in analyzing a random or targeted removal of farms from the network. These models and the results obtained with them can therefore not be compared directly with our findings.

### Gross Margin Analysis

The sensitivity analysis showed that the economic model was most sensitive to alterations in the parameters associated with production losses. The GMA revealed a mean economic impact of 56 Euros per TI dairy cow, 8 Euros per heifer and 5 Euros per calf and thus falls within the range of previous studies: Two worldwide reviews quote the economic impact of BVD as 0.4–585 Euros (1) or 0–621 Euros per animal (2). Losses per cow and year were estimated at 56-133 Euros for France (51), 9.2 Euros for Norway (37) and 75.5–79.1 Euros for Switzerland (14). In Bavaria, losses were estimated at 40 Euros per TI in a lactating cow, 25 Euros per TI in a non-lactating cow and 25 Euros per young stock or heifer (52). In the Netherlands, the production losses per milking cow due to BVD were estimated to range from 19 to 384 Euros per milking cow, with 72 Euros as the most likely value (53). However, these costs included a biannual vaccination of all cows in the herd against BVD and further actions that had to be taken by a farmer to obtain a BVD-free status for the herd. In general, reproduction losses associated with BVD may vary greatly and it is difficult to compare them for different population sizes, herd, and animal-specific conditions and periods.

### Limitations

The disease spread model (DSM) was designed to model the disease spread via animal trade. Each farm can sell and buy from all other farm, which might promote the spread of the virus and thus lead to an overestimation of the number of affected farms. Also, the model simulates the continuous influx of a certain proportion of PI (2% of imported animals). The assumed influx represents a worst-case scenario, which may not necessarily reflect the true current situation. However, Germany is not entitled to additional guarantees of BVD-freedom if cattle are imported from other EU member states, so an influx of PI animals through cattle trade with farmers in other EU member states is possible. If and when Germany may achieve freedom from BVD with a smaller number of imported PI animals will have to be analyzed in a future study. Moreover, virus spread by people (e.g., animal traders, veterinarians, farmers) is not taken into account in the model, which might lead to an underestimation of the PI incidence. We also had to make some simplifications in the model: We did not consider different (combinations of) BVD vaccines, but simulated only the use of a single vaccine with “standard” efficacy. We also assumed that PI calves do not receive maternal antibodies. This should have no influence on the model, since there was only a small number of PI calves. On the other hand, serological testing of calves with maternal antibodies leads to false-positive test results and consequently to the virological testing of the whole herd with a consequence of a massive overestimation of the number of tests.

A sensitivity analysis was carried out for several parameters, e.g., population size, transmission rate, time lag until retesting, vaccination efficacy (29, 30). When we compared the output of the model with the data available from the German cattle trade database, we observed similar trade patterns and age distributions. Socio-economic or animal welfare aspects as well as trade benefits that may arise from a BVDV-free status as well as possible future developments in European legislation on BVD control were beyond the scope of this study. Increased biosecurity in terms of quarantine in combination with testing imported animals in quarantine in the farm of destination may decrease the risk of virus spread. The PI prevalence might be reduced accordingly in all scenarios that include control measures. However, enhanced biosecurity measures were not taken into account in our model.

In conclusion, we modeled the spread of BVDV with and without control measures and calculated the economic impact of the disease and its control using data from Germany. Our analysis showed that within the given limitations, only scenarios with a combination of testing and compulsory vaccination will lead to eradication. However, these scenarios are not beneficial from an economic perspective. The currently implemented eradication program is likely to reduce the PI prevalence to a very low value close to 0 % at a reasonable cost-benefit ratio.

## Author Contributions

JG, FC, and CP contributed conception and design of the study. JG, PB, JB, and PH developed the agent-based model. JG, CP, and FC developed and run the gross margin analysis. JG developed the animal valuation model (AVM). JG and CP developed the economic model. CP and JG wrote the first draft of the manuscript. All authors contributed to manuscript revision, read and approved the submitted version.

### Conflict of Interest

The authors declare that the research was conducted in the absence of any commercial or financial relationships that could be construed as a potential conflict of interest.

## Footnotes

^1^**Control** means, a pre-defined control measure is carried out (e.g., culling, trade restriction, quarantine), if a BVDV positive animal is detected. **Monitoring** is a systematic measurement of animal health, where a positive result does not necessarily lead to action.
