# Supplementary material for: An Epidemiological and Economic Simulation Model to Evaluate Strategies for the Control of Bovine Virus Diarrhea in Germany
Source: Front Vet Sci. 2019 Nov 19;6:406. doi: 10.3389/fvets.2019.00406 (PMC6877714; doi:10.3389/fvets.2019.00406)
Supplement: Supplementary file 1 [file Data_Sheet_1.pdf]

An epidemiological and economic simulation model to evaluate strategies for the control of bovine virus diarrhea in Germany

Supplementary material

Table S1: Input parameters and distributions used in the Gross margin analysis (GMA)........ 2

Table S2: Input parameters used in the animal valuation model to estimate the market value of PI animals (cows, young stock, calves). 5

Table S3: Input parameters and equations used in the economic model. 6

Table S4: Equations used in the benefit-cost analysis 11

Table S5: Probability of transient BVDV infections per pregnancy stage (and overall probability). 12

Table S6: Prolongation of the calving interval (Ci) due to a transient BVDV infection on the foetus 12

Table S7: Results of the economic model: Mean total costs (million Euros), thereof direct and indirect costs (million Euros). 13

Table S1: Input parameters and distributions used in the Gross margin analysis (GMA).

**Parameter**	**Unit**	**Notation**	**Calculation/ value/ distribution**	**References/ comments**
**G**ross **M**argin for a dairy farm	Euro	GM	$GM = R - VC$	
**R**evenues	Euro	R	$R = R_{milk} + R_{ani} + R_{man}$	
**R**evenues for selling **milk**	Euro	R_{milk}	$R_{milk} = my * ms * mp$	
annual **m**ilk **y**ield healthy animal	kg/cow	my	$my = rnorm(10000, 7087.25, 136.11)$	Normal distribution based on data from Federal statistical office (Destatis) on milk yield on district level 2011-2015
**i**nfluence BVD infection on **m**ilk **y**ield	kg/cow	imy	$imy = my * rlnorm(10000, log(0.01), 0.008)$	The decrease in milk yield was estimated at about 70 liters.
annual **m**ilk **y**ield **BVD**V infected animal	kg/cow	my_{BVD}	$my_{BVD} = my * \frac{(Ci - 60)}{(Ci_{BVD} - 60)} - imy$	The annual milk yield for an infected animal includes the reduction of the milk yield due to acute symptoms as well as the reduction caused by a prolonged calving interval
proportion of **m**ilk **s**old	%		$ms = 95$	Destatis: milk sold on district level 2011-2015
**M**ilk **p**rice	Euro/kg	Mp	$mp = dUnif(0.289082, 0.360424)$	Uniform distribution based on data from Federal Ministry of Food and Agriculture; BLE: monthly average milk prices on federal state level
**R**evenues for selling **ani**mals	Euro	R_{ani}	$R_{ani} = R_{calf} + R_{cow}$	
**R**evenues from **calf** sale	Euro	R_{calf}	$R_{calf} = CC * p_{mc} * r + CC * p_{fc} * (1 - r)$	
number of **c**alves per **c**ow	n	CC	$CC = \frac{365}{Ci} - \frac{365}{Ci * cm_y}$	
**p**rice **m**ale **c**alf	Euro	p_{mc}	$p_{mc} = \mathrm{dTri}(80.94, 121.41, 105.22)$	Betriebsplanung Landwirtschaft 2014/15, KTBL: price for 14 days old calves
**p**rice **f**emale **c**alf	Euro	p_{fc}	$p_{fc} = \mathrm{dTri}(30.35, 60.70, 44.52)$	
male/female **r**atio	ratio	R	$r = 0.5$	Estimated
**R**evenues from **cow** sale	Euro	R_{cow}	$R_{cow} = (pC * wC - mC) * (1 - CM) * RR$	
**p**rice slaughter **c**ow	Euro/kg	pC	$\mathrm{pC} = 2.05$	Bavarian State Research Centre for Agriculture, *Bayerische Landesanstalt für Landwirtschaft* https://www.stmelf.bayern.de/idb/vergleiche.html
average **w**eight of a slaughter **c**ow	kg	wC	$\mathrm{wC} = 326.46$	
**m**arketing costs of a **c**ow	Euro	mC	$\mathrm{mC} = 21$	
**c**ow **m**ortality	ratio	CM	$\mathrm{CM} = 0.054$	
**r**eplacement **r**ate	ratio	RR	$\mathrm{RR} = 0.33$	Bayerische Landesanstalt für Landwirtschaft, https://www.stmelf.bayern.de/idb/vergleiche.html

**c**alving **i**nterval healthy animal	days	Ci	$\mathrm{Ci} = \mathrm{dTri}(376, 417, 458)$	Triangle distribution based on data from HI Tier
**c**alving **i**nterval with **BVD** infection	days	Ci_{BVD}	$Ci_{BVD} = Ci + iCi_{BVD}$	
**c**alf **m**ortality	ratio	cm	$\mathrm{cm} = 0.1$	fixed based on data from Bayerische Landesanstalt für Landwirtschaft, https://www.stmelf.bayern.de/idb/vergleiche.html
**i**nfluence of BVD infection on **c**alf **m**ortality	ratio	icm	$\mathrm{icm} = 0.1$	
**c**alf **m**ortality with **BVD** infection	ratio	cm_{BVD}	$\mathrm{cm}_{BVD} = \mathrm{cm} + \mathrm{icm}$	
**i**ncreased **c**alving **i**nterval (additional number of days) in case of transient **BVD** infection	days	iCi_{BVD}	$iCi_{BVD} = lnorm(log(10.5), log(2))$	iCi_{BVD} is assumed as log normal distribution to increase about 13 days on average. Estimated based on (Burgstaller et al., 2016; Viet et al., 2004).
**R**evenues for selling **man**ure	Euro	R_{man}	$R_{man} = 111.1$	Fixed (based on data of the Bavarian State Research Centre for Agriculture, *Bayerische Landesanstalt für Landwirtschaft;* https://www.stmelf.bayern.de/idb/vergleiche.html
**V**ariable **C**osts	Euro	VC	$VC = VCR_y + VCfe_y + VCcr_y + VCvet_y + VCbio_y + VCins_y + VCmach_y + VCmisc_y$	
**V**ariable **C**osts for **r**estocking per **y**ear	Euro	VCR_y	$VCR_y = (p_h + m_h) * RR$	
**p**rice for a **h**eifer	Euro	p_h	$p_h = 1{,}950$	Fixed (based on data of the Bavarian State Research Centre for Agriculture, *Bayerische Landesanstalt für Landwirtschaft;* https://www.stmelf.bayern.de/idb/vergleiche.html
**m**arketing costs for a **h**eifer	Euro	m_h	$m_h = 33$	
**V**ariable **C**osts for **fe**ed per year	Euro	$VCfe_y$	$VC_{fe} = \frac{my - my_f}{ef_{con}} * p_{con}$	
**m**ilk **y**ield from **f**orage	kg	my_f	$my_f = \frac{(f_u * f_q - bmr)}{mf}$	
**F**orage **u**ptake per year	kg	f_u	$f_u = 4562.5$	Fixed (based on data of the Bavarian State Research Centre for Agriculture, *Bayerische Landesanstalt für Landwirtschaft;* https://www.stmelf.bayern.de/idb/vergleiche.html
**F**orage **q**uality	factor	f_q	$f_q = 5.9$	
**m**ilk per kg **f**orage	factor	mf	$mf = 3.3$	
**b**asal **m**etabolic **r**ate	MJ	bmr	$bmr = 15300$	
**ef**ficiency of **con**centrate use	l /kg	ef_{con}	$ef_{con} = rtri(niteration, 1.7,2,1.9)$	

**p**rice for feed **con**centrates	Euro/kg	p_{con}	$p_{con} = 0.28$	Fixed (based on data of the Bavarian State Research Centre for Agriculture, *Bayerische Landesanstalt für Landwirtschaft)*
**V**ariable **C**osts for **c**alf **r**earing per **y**ear	Euro	$VCcr_y$	$VCcr_y = 57.66$	Fixed (based on data of the Bavarian State Research Centre for Agriculture, *Bayerische Landesanstalt für Landwirtschaft;* https://www.stmelf.bayern.de/idb/vergleiche.html
**V**ariable **C**osts for **vet**erinary treatment per **y**ear	Euro	$VCvet_y$	$VCvet_y = 165$	
**V**ariable **C**osts for **wa**ter and electricity per **y**ear	Euro	$VCwa_y$	$VCwa_y = 75.63$	
**V**ariable **C**osts for artificial **ins**emination per **y**ear	Euro	$VCins_y$	$VCins_y = 9.41$	
**V**ariable **C**osts for **mach**ines per **y**ear	Euro	$VCmach_y$	$VCmach_y = 58.82$	
**V**ariable **C**osts, **misc**elaneous (dues) per **y**ear	Euro	$VCmisc_y$	$VCmisc_y = 36.13$	
**D**irect **C**osts per **TI** animal	Euro	DC_{TI}	$DC_{TI} = GM_h - GM_{TI}$	The GM for a healthy animal (GM_h) and TI animal (GM_{TI}) are calculated using equation 1

Table S2: Input parameters used in the animal valuation model to estimate the market value of PI animals (cows, young stock, calves).

**Parameter**	**Unit**	**Notation**	**Calculation/ value/ distribution**	**References/ comments**
market **v**alue of a **c**ow	Euro	vc_y	$vc_y = B \times (1 + bp + mp + ib + da + p + c) + m$	based on the data provided by the chamber of agriculture of North-Rhine-Westphalia
**B**asic value	Euro	B		
depreciation for not being registered in a **b**reeding **p**rogram	per cent	bp	$bp = -10$	
depreciation for not being included in a **m**ilk inspection **p**rogram	per cent	mp	$mp = -30$	According to http://milchwirtschaft.de/, in 2014 88% of all German dairy cows were included in the milk inspection program (Milchleistungsprüfung)
depreciation for not being free of **i**nfectious **b**ovine rhinotracheitis	per cent	ib	$ib = -10$	
**d**epreciation according to the **a**ge of the animal	per cent	da	$da = -10 - \ -40$	We used 1-month age groups. According to HIT, the age distribution was as follows: 65 % of the animals were 3-6 years; 15 % 6-7 years; 7% 7-8 years; 5% 8-9 years; 0.07 >9 years.
premium for **p**regnancy status	per cent	p	$p = \begin{cases} 0, & < 4 \text{ months} \\ 4, & \geq 4 \text{ months} \\ 8, & \geq 6 \text{ months} \end{cases}$	we assumed that a cow will gain a bonus of 0.04 for 4 month per year (4-7 months pregnant) and 0.08 for two month per year (8-9 months pregnant).
premium for general body **c**ondition	per cent	c	$c = 4$	For each point above a body condition of 80
premium for **m**ilk protein	Euro	m	$m = \begin{cases} 0, & = \text{average} \\ 4 \ per\ kg, & > \text{average} \\ -4 \ per\ kg, & < \text{average} \end{cases}$	4 € for each kg above-average, -4 € for each kg below-average
market **v**alue of a calf or **y**oung stock per **y**ear	Euro	vy_y	$vy_y = \ 0.2 * (B * (1 + c) + m) + \frac{(B * (1 + c) + m) - 0{,}2 * (B * (1 + c) + m)}{afc} \times a$	
**a**ge at **f**irst **c**alving of the mother	months	afc	$afc = 28$	fixed value
**a**ge of the animal	months	a		

Table S3: Input parameters and equations used in the economic model.

Parameter	Notation	Calculation/ value/ distribution	References/ comments
**T**otal **C**osts of BVD for the 20-year period 2011-2030 in each **s**cenario	**CT_s**	$CT_s = \sum_{y=2011}^{2030} DC_{y,s} + IC_{y,s}$	
**T**otal **C**osts of BVD per **y**ear in each **s**cenario	**$CT_{y,s}$**	$CT_{y,s} = DC_{y,s} + IC_{y,s}$	
**D**irect **C**osts of BVD per **y**ear in each **s**cenario	**$DC_{y,s}$**	$DC_{y,s} = DCPI_{y,s} + DCTI_{y,s}$	
**D**irect **C**osts incurred by **PI**s per **y**ear in each **s**cenario	**$DCPI_{y,s}$**	$DCPI_{y,s} = DCPIc_{y,s} + DCPIy_{y,s} + DCPIh_{y,s} + DCPIC_{y,s}$	Includes the market value of animals that die due to a persistent BVDV infection (e.g. due to Mucosal disease), disposal costs of these dead animals, and veterinary costs.
**D**irect **C**osts PI **c**alves	$DCPIc_{y,s}$	$DCPIc_{y,s} = n_{PIcy,s} * C_{PI}$	
**D**irect **C**osts PI **y**oung cattle	$DCPIy_{y,s}$	$DCPIy_{y,s} = n_{PIyy,s} * C_{PI}$	
**D**irect **C**osts PI **h**eifers	$DCPIh_{y,s}$	$CDPIh_{y,s} = n_{PIhy,s} * C_{PI}$	
**D**irect **C**osts PI **C**ows	$DCPIC_{y,s}$	$DCPIC_{y,s} = n_{PICy,s} * C_{PI}$	At the level of the national livestock sector, studies indicated a loss due to BVDV under endemic conditions of € 15-20 per cow present (Anonymus, 2001)
**n**umber of PI **c**alves, **y**oung stock, **h**eifers, and **c**ows respectively per **y**ear in each **s**cenario	$n_{PIcy,s}$ $n_{PIyy,s}$ $n_{PIhy,s}$ $n_{PICy,s}$		Calculated in the DSM (using 1-month age groups). According to the age, animals were categorized as calves (0-6 months), young cattle (7-18 months), heifers (19-28 months), and cows (older than 29 months).
average direct **c**osts per **PI** animal	C_{PI}	$C_{PI} = vet_{PI} + pd_{PI}$	
costs of **vet**erinary treatment of a **PI** animal	vet_{PI}	Triangle distribution (27.50; 36.10; 72.70)	Include travel costs, clinical examination and advice, one injection/ infusion, and antibiotic treatment. As per current legal framework (German veterinary fee schedule, *Gebührenordnung für Tierärzte*), veterinarians may charge travel costs of 2.30 € per double km (minimum of 8,60 €). Examination and advice amount to 11.46 to 34.38 €, injection/ infusion to 3.44 to 10.32, and antibiotic treatment to 4-5 €.

costs and lost value through **p**remature **d**eath of a **PI** animal	pd_{PI}	$pd_{PI} = v_c + d_c$	
market **v**alue of a dead animal	v_c		Calculated in the animal valuation model
**d**isposal costs (calf, young stock, cow)	d_c, d_y, d_C	calf 7.80 €; young stock, heifer 35.10 €; cow 68.90 €	Fixed values
**D**irect **C**osts incurred by **TI**s per **y**ear in each **s**cenario	$\mathbf{DCTI_{y,s}}$	$DCTI_{y,s} = DCTIc_{y,s} + DCTIy_{y,s} + DCTIh_{y,s} + DCTIC_{y,s}$	Include production losses
**D**irect **C**osts **TI c**alves	$DCTIc_{y,s}$	$DCTIc_{y,s} = n_{TIcy,s} * DCTIa_c$	
**D**irect **C**osts **TI y**oung cattle	$DCTIy_{y,s}$	$DCTIy_{y,s} = n_{TIyy,s} * DCTIa_y$	
**D**irect **C**osts **TI h**eifers	$DCTIh_{y,s}$	$DCTIh_{y,s} = n_{TIhy,s} * DCTIa_h$	
**D**irect **C**osts **TI c**ows	$DCTIC_{y,s}$	$DCTIC_{y,s} = n_{TICy,s} * DCTIa_C$	
**n**umber of TI **c**alves, **y**oung cattle, **h**eifers, and **c**ows respectively per **y**ear in each **s**cenario	$n_{TIcy,s}$ $n_{TIyy,s}$ $n_{TIhy,s}$ $n_{TICy,s}$		Calculated in the DSM (using 1-month age groups). According to the age, animals were categorized as calves (0-6 months), young cattle (7-18 months), heifers (19-28 months), and cows (older than 29 months).
**D**irect **C**osts **TI**: **a**verage production losses incurred per **c**alf	$DCTIa_c$	Uniform distribution (0;10)	
**D**irect **C**osts **TI**: **a**verage production losses incurred per **y**oung cattle	$DCTIa_y$	Uniform distribution (0;10)	
**D**irect **C**osts **TI**: **a**verage production losses incurred per **h**eifer	$DCTIa_h$	Resampling from GMA results	Calculated in the GMA for a heifer (Resampling done randomly with replacement).
**D**irect **C**osts **TI**: **a**verage production losses incurred per **c**ow	$DCTIa_C$	Resampling from GMA results	Calculated in the GMA for a cow (Resampling done randomly with replacement).
**I**ndirect **C**osts of BVD per **y**ear in each **s**cenario	$\mathbf{IC_{y,s}}$	$IC_{y,s} = ICdi_{y,s} + ICva_{y,s} + ICps_{y,s} + ICtr_{y,s}$	
**I**ndirect **C**osts incurred by **di**agnostic measures per **y**ear in each **s**cenario	$\mathbf{ICdi_{y,s}}$	$ICdi_{y,s} = ICdidt_{y,s} + ICdidb_{y,s} + ICindb_{y,s}$	
**I**ndirect **C**osts incurred by antigen analysis (**di**rect detection) by **t**issue sampling (ear tag) per **y**ear in each **s**cenario	$ICdidt_{y,s}$	$ICdidt_{y,s} = (n_{t1y,s} * C_{t1}) + (n_{t2y,s} * C_{t2})$	

**n**umber of animals **t**ested (ear tag, first (**1**) application)	$n_{t1y,s}$		calculated in the DSM
**C**osts antigen detection ear tags, first (**1**) analysis	C_{t1}	$C_{t1} = c_{t1} + c_p/5 + c_{sh} + c_{ELI1} + c_c$	
**c**osts diagnostic ear **t**ags, first (**1**) application	c_{t1}	Triangle distribution (3;4;5)	According to Landeskontrollverband Baden-Württemberg: 1,12 € for two ear tags; 0.51 € shipping; 3.60 € laboratory testing; 1.80 € diagnostic material (Anonymus, 2010)
**c**ost ear tag **p**liers	c_p	Triangle distribution (15; 20; 25) / 5	Costs for ear tag pliers according to Sächsischer Landeskontrollverband; We accounted for one fifth of the costs, since pliers can be used for several animals.
**c**osts **sh**ipping	c_{sh}	Triangle distribution (0.1; 0.2; 0.5)	
**c**osts laboratory analysis (erns **ELI**SA), first (**1**) test	c_{ELI1}	Triangle distribution (3.5; 4.5; 5.5)	
**c**osts **c**ommunication of the test result	c_c	Triangle distribution (0.2; 0.4; 0.8)	
**n**umber of animals **t**ested (ear tag, second (**2**) application)	$n_{t2y,s}$		
**C**osts antigen detection ear **t**ags, second (**2**) analysis	C_{t2}	$C_{t2} = c_{t2} + c_{ELI2}$	
**c**osts diagnostic ear **t**ags, second (**2**) application	c_{t2}	Uniform distribution (1.45; 2)	
**c**osts laboratory analysis (erns **ELI**SA), second (**2**) test	c_{ELI2}	Fixed value (15)	
**I**ndirect **C**osts incurred by antigen analysis (**di**rect **d**etection) by **b**lood sampling per **y**ear in each **s**cenario	$ICdidb_{y,s}$	$ICdidb_{y,s} = (n_{dby,s} * C_{db}) + (n_{fdby,s} * C_{fb})$	
**n**umber of animals tested (**d**irect detection) by **b**lood (antigen)	$n_{dby,s}$		calculated in the DSM
**C**osts **d**irect detection by **b**lood (antigen) per animal	C_{db}	$C_{db} = c_{bs} + c_{PCR}$	
**C**osts **b**lood **s**ampling	C_{bs}	Triangle distribution (3;7;10)	According to the official scale of fees for veterinarians (Gebührenordnung für Tierärzte)
**C**osts laboratory analysis (**PCR**)	c_{PCR}	Triangle distribution (5;8;11)	

**n**umber of **f**arms sampled (for **d**irect detection in **b**lood samples)	$n_{fdby,s}$		calculated in the DSM
**C**osts per **f**arm for **b**lood sampling	C_{fb}	$C_{fb} = c_{hf} + c_s + c_{sh} + c_c$	
**c**osts **h**erd **f**ee	c_{hf}	Triangle distribution (17;19;23)	Herd fee charged for blood sampling by veterinarians are … Euros per callout and includes travel costs and veterinary advice
**c**osts **s**ample handling	c_s	Fixed (2)	
**c**osts sample **sh**ipping	c_{sh}	Triangle distribution (0.1; 0.2; 0.5)	
**c**osts **c**ommunication of the test result	c_c	Triangle distribution (0.2; 0.4; 0.8)	
**I**ndirect **C**osts incurred by antibody testing (**in**direct **d**etection) by **b**lood sampling per **y**ear in each **s**cenario	$ICindb_{y,s}$	$ICindb_{y,s} = (n_{iby,s} * C_{ib}) + (n_{fiby,s} * C_{fb})$	
**n**umber of animals tested (**i**ndirect detection) by **b**lood (antibodies)	$n_{iby,s}$		calculated in the DSM
**C**osts antibody testing (**i**ndirect detection) by **b**lood per animal	C_{ib}	$C_{ib} = c_{bs} + c_{ELISA}$	
**c**osts laboratory analysis (**ELISA**)	c_{ELISA}	Triangle distribution (8; 9; 10)	
**n**umber of **f**arms sampled (for **i**ndirect detection in **b**lood)	$n_{fiby,s}$		calculated in the DSM
**I**ndirect **C**osts incurred by **va**ccination per **y**ear in each **s**cenario	$ICva_{y,s}$	$ICva_{y,s} = (n_{vafy,s} * C_{vaf}) + (n_{vaiy,s} * n_{vi} * c_{va} + c_{vac}$[illegible]	
**n**umber of **va**ccinated **f**arms	$n_{vafy,s}$		calculated in the DSM
**C**osts for **va**ccination, **f**arm level	C_{vaf}	$C_{vaf} = c_{hf}$	Herd fee charged for vaccination by veterinarians are … Euros per callout and includes travel costs and veterinary advice
**n**umber of **va**ccinated **a**nimals	$n_{vaiy,s}$		calculated in the DSM
**n**umber of **v**accinations (**i**mmunisations) per animal	n_{vi}	Triangle distribution (1.2;1.25;1.33)	
**c**osts for one **va**ccine	c_{va}	Triangle distribution (3;4;5)	Vaccine: (Pape, 2000)
**c**osts for **vacc**inating one animal	c_{vacc}	Triangle distribution (1;1.4;1.8)	
**I**ndirect **C**osts incurred by **p**reventive **s**laughter of PIs per **y**ear in each **s**cenario	$ICps_{y,s}$	$ICps_{y,s} = (nsPIc_{y,s} * v_{PIc}) + (nsPIy_{y,s} * v_{PIy}) + (nsPIC_{y,s} * v_{PIC})$	

**n**umber of preventively **s**laughtered **PI c**alves (0-6 months), **y**oung cattle (6-24 months), **c**ows (>24 months)	$nsPIc_{y,s}$<br>$nsPIy_{y,s}$<br>$nsPIC_{y,s}$		calculated in the DSM
compensation (**v**alue) for preventive slaughtering a **PI c**alf	v_{PIc}	Uniform distribution (18;75)	Based on the compensation for preventive slaughtering ("Merzungsbeihilfe") provided by the animal health insurances (Tierseuchenkassen). Compensation depends among other factors, on the breed, gender and age of the animal.
Compensation (**v**alue) for preventive slaughtering a **PI y**oung cattle	v_{PIy}	Uniform distribution (3;150)	
Compensation (**v**alue) for preventive slaughtering a **PI C**ow	v_{PIC}	Uniform distribution (150;400)	
**I**ndirect **C**osts incurred by **t**rade **r**estrictions per **y**ear in each **s**cenario	$ICtr_{y,s}$	Triangular distribution (100;115;118)	Based on (Anonymus, 2016), we assumed that within 40 days of quarantine, each affected farm would want to move three pregnant and three non-pregnant animals. This implies the following costs for three pregnant and three non-pregnant animals respectively: travel 10 €, taking blood samples 10 €, handling and shipping samples 9 €, laboratory analysis 30 € (3x10 €). Hence, the movement ban would result in 118 € (2x59 €) additional veterinary costs per affected premise.

Anonymus, 2001. Position paper of the EU Thematic network on control of bovine viral diarrhoea virus (BVDV).

Anonymus, 2010. Antwort auf die Kleine Anfrage Drucksache 17/3263 Ohrstanz-Gewebeprobe bei Rindern im Rahmen der BVD-Impfung.

Anonymus, 2016. Zweite Verordnung zur Änderung der BVDV-Verordnung. Drucksache 200/16 vom 20.04.16.

Burgstaller, J., Obritzhauser, W., Kuchling, S., Kopacka, I., Pinior, B., Köfer, J., 2016. The effect of bovine viral diarrhoea virus on fertility in dairy cows: two case-control studies in the province of Styria, Austria. Berl Münch Tierärztl Wochenschr 129, 103-110.

Pape, K.-R., 2000. BVD-Bekämpfung: Worüber die Experten streiten. Top agrar 6, 18-21.

Viet, A.F., Fourichon, C., Seegers, H., Jacob, C., Guihenneuc-Jouyaux, C., 2004. A model of the spread of the bovine viral-diarrhoea virus within a dairy herd. Prev Vet Med 63, 211-236.

Table S4: Equations used in the benefit-cost analysis

Parameter	Notation	Equation
**B**enefit-**C**ost **R**atio in each **s**cenario	BCR_s	$BCR_s = \frac{\sum_{y=2011}^{2030} B_{y,s}}{\sum_{y=2011}^{2030} IC_{y,s}}$
**P**resent **V**alue **B**enefit	PVB_s	$PVB_s = \sum_{y=2011}^{2030} \frac{B_{y,s}}{(1+r)^{(y-2011)}}$
**B**enefit per **y**ear	$B_{y,s}$	$B_{y,s} = DC_{y,s1} - DC_{y,s}$
Interest **r**ate	r	$r = 3\%$
**P**resent **V**alue **I**ndirect **C**osts	$PVIC_s$	$PVIC_s = \sum_{y=2011}^{2030} \frac{IC_{y,s}}{(1+r)^{(y-2011)}}$
**N**et **v**alue	NV_s	$NV_s = \sum_{y=2011}^{2030} B_{y,s} - \sum_{y=2011}^{2030} IC_{y,s}$
**N**et **p**resent **v**alue	NPV_s	$NPV_s = \sum_{y=2011}^{2030} \frac{B_{y,s}}{(1+r)^{(y-2011)}} - \sum_{y=2011}^{2030} \frac{IC_{y,s}}{(1+r)^{(y-2011)}}$

Table S5: Probability of transient BVDV infections per pregnancy stage (and overall probability).

effect	Pregnancy stage								Post partum	
	**days 0 - 70**		**days 71 - 120**		**days 121 - 180**		**days 181 - 285**		**days 286 - 385**	
birth of a PI calf	0.90	(0.16)	0.45	(0.06)	0.00	(0.00)	0.00	(0.00)	0.00	(0.00)
abortion. stillbirth	0.10	(0.02)	0.15	(0.02)	0.20	(0.03)	0.05	(0.01)	0.00	(0.00)
congenital defects. growth retardation	0.00	(0.00)	0.15	(0.02)	0.25	(0.04)	0.15	(0.04)	0.00	(0.00)
Immune	0.00	(0.00)	0.25	(0.03)	0.55	(0.09)	0.80	(0.22)	0.00	(0.00)
no effect	0.00	(0.00)	0.00	(0.00)	0.00	(0.00)	0.00	(0.00)	1.00	(0.26)
**total**	**1.00**	**(0.18)**	**1.00**	**(0.13)**	**1.00**	**(0.16)**	**1.00**	**(0.27)**	**1.00**	**(0.26)**

Table S6: Prolongation of the calving interval (Ci) due to a transient BVDV infection on the foetus

	**days 0 - 70**	**days 71 - 120**	**days 121 - 180**	**days 181 - 285**	**days 286 - 385**	**total average prolongation of the Ci (days)**
average prolongation of the Ci (days)	35	95	150	233	335	
probability of outcome of abortion. stillbirth (see table S5)	0.02	0.02	0.03	0.01	0.00	
prolongation of the Ci (days)	0.64	1.85	4.68	3.17	0.00	**10.33**

Table S7: Results of the economic model: Mean total costs (million Euros), thereof direct and indirect costs (million Euros).

scenario	costs	2011	2012	2013	2014	2015	2016	2017	2018	2019	2020	2021	2022	2023	2024	2025	2026	2027	2028	2029	2030
**sc1**	total costs	129	129	132	122	122	120	118	117	117	115	113	108	106	105	103	104	99	100	100	100
	direct costs	129	129	132	122	122	120	118	117	117	115	113	108	106	105	103	104	99	100	100	100
**sc 2**	total costs	195	146	117	109	99	92	87	86	87	88	86	84	83	82	82	82	83	82	81	80
	direct costs	105	55	31	26	19	14	11	10	11	11	9	8	7	6	6	7	7	6	5	5
	indirect costs	91	91	86	83	80	78	77	76	76	77	76	76	76	76	76	75	76	76	75	75
**sc 3**	total costs	195	146	117	109	99	91	87	86	87	86	85	83	81	81	81	79	79	80	79	79
	direct costs	105	55	31	26	19	13	10	10	10	10	9	7	6	5	5	4	4	4	4	4
	indirect costs	90	91	86	83	80	78	77	76	76	77	76	76	75	75	75	75	75	75	75	75
**sc 4**	total costs	195	146	117	108	99	91	79	53	78	71	46	37	39	39	36	35	36	38	35	36
	direct costs	104	56	31	26	19	13	17	53	78	71	46	37	39	39	36	35	36	38	35	36
	indirect costs	90	90	86	83	80	78	62	0	0	0	0	0	0	0	0	0	0	0	0	0
**sc 5**	total costs	195	146	117	109	99	92	87	92	109	118	125	132	137	138	138	138	138	139	138	138
	direct costs	105	55	31	26	19	13	10	10	10	5	1	0	0	0	0	0	0	0	0	0
	indirect costs	91	91	86	83	80	78	77	82	99	113	123	132	137	138	138	138	138	139	138	138
**sc 6**	total costs	195	147	118	109	99	91	89	310	283	256	238	221	209	200	195	190	184	177	174	171
	direct costs	104	56	32	26	19	13	12	10	11	11	11	10	8	7	6	6	5	5	4	5
	indirect costs	91	91	86	83	80	78	77	300	272	244	227	211	201	193	189	184	179	172	170	166
**sc 7**	total costs	195	146	118	109	99	92	89	178	162	152	141	130	122	119	116	113	112	109	105	104
	direct costs	104	56	32	26	19	13	12	10	10	11	11	9	7	6	6	6	6	5	4	4
	indirect costs	91	91	86	83	80	78	77	168	152	141	130	121	115	113	110	108	107	104	101	100
**sc 8**	total costs	195	146	117	109	99	91	87	297	378	437	491	516	527	534	533	534	533	535	535	534
	direct costs	104	56	31	26	19	13	10	10	8	3	1	0	0	0	0	0	0	0	0	0
	indirect costs	90	90	86	83	80	78	77	287	370	433	491	516	527	534	533	534	533	535	535	534
**sc 9**	total costs	195	146	118	109	99	91	85	116	124	131	138	146	150	151	151	151	151	151	151	151
	direct costs	105	55	31	26	19	13	9	10	8	3	1	0	0	0	0	0	0	0	0	0
	indirect costs	90	91	86	83	80	78	76	106	116	127	137	145	150	151	151	151	151	151	151	151
**sc 10**	total costs	195	146	117	109	99	91	56	243	222	183	150	134	134	136	130	116	106	105	105	104
	direct costs	104	56	31	26	19	13	14	26	32	18	8	6	10	14	13	8	5	6	8	9

scenario	costs	2011	2012	2013	2014	2015	2016	2017	2018	2019	2020	2021	2022	2023	2024	2025	2026	2027	2028	2029	2030
	indirect costs	91	91	86	83	80	78	42	217	190	165	142	128	124	122	117	108	101	99	96	95
**sc 11**	total costs	194	146	117	109	99	91	58	55	94	99	84	81	82	81	76	72	71	69	68	66
	direct costs	104	55	31	26	19	13	16	50	71	62	36	23	19	18	13	8	7	5	4	3
	indirect costs	91	91	86	83	80	78	42	5	23	37	49	58	63	64	63	63	63	64	64	63
**sc 12**	total costs	194	145	117	108	99	91	87	59	63	81	84	76	78	75	73	70	68	67	66	65
	direct costs	104	56	31	26	19	13	10	15	41	44	36	19	15	12	10	7	5	4	3	2
	indirect costs	90	90	86	83	80	78	77	44	22	37	48	57	62	63	63	63	63	63	63	63
**sc 13**	total costs	195	146	117	109	99	91	54	62	142	155	153	156	158	159	159	158	158	159	158	158
	direct costs	104	56	31	26	19	13	11	16	27	16	4	1	0	0	0	0	0	0	0	0
	indirect costs	90	90	86	83	80	78	43	47	115	139	149	155	158	159	159	158	158	159	158	158
